# Individual and Neighborhood Socioeconomic Status and Healthcare Resources in Relation to Black-White Breast Cancer Survival Disparities

**DOI:** 10.1155/2013/490472

**Published:** 2013-02-20

**Authors:** Tomi F. Akinyemiju, Amr S. Soliman, Norman J. Johnson, Sean F. Altekruse, Kathy Welch, Mousumi Banerjee, Kendra Schwartz, Sofia Merajver

**Affiliations:** ^1^Department of Epidemiology, Columbia University Mailman School of Public Health, New York, NY 10032, USA; ^2^Department of Epidemiology, College of Public Health, University of Nebraska Medical Center, Omaha, NE 68198, USA; ^3^U.S. Census Bureau-National Longitudinal Mortality Survey, 4700 Silver Hill Rd., Suitland, MD 20746, USA; ^4^Cancer Statistics Branch, Division of Cancer Control and Population Sciences, National Cancer Institute, Bethesda, MD 20892, USA; ^5^Center for Statistical Consulting and Research, University of Michigan, Ann Arbor, MI 48109, USA; ^6^Department of Biostatistics, University of Michigan School of Public Health, Ann Arbor, MI 48109, USA; ^7^Department of Family Medicine and Public Health Sciences and Barbara Ann Karmanos Institute, Wayne State University School of Medicine, Detroit, MI 48201, USA; ^8^Department of Internal Medicine, University of Michigan Medical School, Ann Arbor, MI 48109, USA; ^9^University of Michigan Center for Global Health, Ann Arbor, MI 48109, USA

## Abstract

*Background.* Breast cancer survival has improved significantly in the US in the past 10–15 years. However, disparities exist in breast cancer survival between black and white women. *Purpose.* To investigate the effect of county healthcare resources and SES as well as individual SES status on breast cancer survival disparities between black and white women. *Methods.* Data from 1,796 breast cancer cases were obtained from the Surveillance Epidemiology and End Results and the National Longitudinal Mortality Study dataset. Cox Proportional Hazards models were constructed accounting for clustering within counties. Three sequential Cox models were fit for each outcome including demographic variables; demographic and clinical variables; and finally demographic, clinical, and county-level variables. *Results.* In unadjusted analysis, black women had a 53% higher likelihood of dying of breast cancer and 32% higher likelihood of dying of any cause (*P* < 0.05) compared with white women. Adjusting for demographic variables explained away the effect of race on breast cancer survival (HR, 1.40; 95% CI, 0.99–1.97), but not on all-cause mortality. The racial difference in all-cause survival disappeared only after adjusting for county-level variables (HR, 1.27; CI, 0.95–1.71). *Conclusions.* Improving equitable access to healthcare for all women in the US may help eliminate survival disparities between racial and socioeconomic groups.

## 1. Introduction

Breast cancer incidence and mortality rates have declined steadily in the US for the past 10–15 years [1–7]. The relative 5-year survival rate for breast cancer overall has also increased in the past decade to 89% [8]. Unfortunately, disparities exist in breast cancer outcomes between racial groups in the US. Although survival has increased for both white and black women over time, the survival increase in black women has been smaller [2]. Five-year relative survival for breast cancer was 90% for white women and 77% for black women between 2001 and 2007 [8, 9]. Several reasons have been suggested for the survival disparity between black and white women, including racial differences in access to and utilization of screening and treatment [10–12], risk factors that are differentially distributed by race [10, 13–15] and socioeconomic status (SES) [14, 16–20], and biological differences such as tumor aggressiveness [21, 22].

The results of research studies assessing the role of SES in racial disparities in breast cancer survival are mixed; some studies reported that racial differences in survival disappear after adjusting for SES [17, 19, 20, 23, 24], while other studies show survival differences persisting after adjustment for SES [13, 14, 17, 20, 25–27]. The conflicting results may be due to the lack of SES information in cancer registries in the US, resulting in insufficient characterization of socioeconomic levels of patients [11, 20, 25, 28]. In addition to SES, differential levels of healthcare resources at the neighborhood level have also been examined in previous studies as potentially contributing to racial disparities in cancer outcomes [29–36]. However, the same limitations exist as with studies of SES effect on racial disparities in breast cancer survival—lack of consistency in which measure of health care resources to use and questions about which geographic level neighborhood differences are measured. 

Defining healthcare access is complicated, mainly because the construct encompasses dimensions of healthcare availability, affordability, acceptability, and accessibility. To ensure that valid inferences are drawn, it is necessary to specify which aspect of healthcare access is being measured. For this study, we focused on the availability of healthcare resources at the county level as our measure of healthcare access. To our knowledge, no other research study has developed a specific measure of neighborhood healthcare access as a predictor of breast cancer survival among blacks and whites in the US, while controlling for individual and neighborhood level SES. This is unique. Other studies attempting to examine the impact of neighborhood effects on breast cancer survival have done so without individual level SES data [26] or did not simultaneously control for healthcare resource availability. The aim of this study was to assess the impact of neighborhood health care resources on white-black disparities in breast cancer survival by adjusting for individual and neighborhood SES. 

## 2. Methods

### 2.1. Data Sources and Analytic Samples

This study utilized the National Cancer Institute's Surveillance Epidemiology and End Results data linked to the US Census Bureau's National Longitudinal Mortality Study database (NLMS). The linked dataset is referred to in this study as (SEER-NLMS). Detailed methodology regarding this dataset has been published elsewhere [13, 37, 38]. In brief, SEER data on cancer incidence, prevalence and survival from 15 participating registries, covering about 25% of the US population [39] was linked with the NLMS data to capture demographic, socioeconomic, and occupation attributes from the Current Population Surveys and the Annual Social and Economic Supplement [38]. A full description of the NLMS is available at the census website (http:/www.census.gov/nlms/). 

Data on county level SES, health care facilities, and health care personnel were obtained from the 2009-2010 Area Resource File [40] which contains over 6,000 variables relating to healthcare, socioeconomic, and environmental characteristics for each county in the US. Data on the number of certified mammography facilities was obtained through a Freedom of Information (FOI) request to the Food and Drug Administration (FDA), the agency in charge of certifications [41]. A list and addresses of all mammography facilities certified in the year 2000 was provided by the FDA. The dataset used in the final analysis was restricted to non-Hispanic black and non-Hispanic white first primary breast cancer cases among women with ages 40 years and older, diagnosed between 1973 and 2003. The analysis was restricted to non-Hispanic black and white women to facilitate the comparison of two racial groups in the US that have shown sustained and consistently high disparities in breast cancer survival. A total of 3511 women fulfilled this criterion: 3283 whites and 228 blacks. We focused the main analysis on women residing in counties that contained at least one black and one white breast cancer patient to ensure that the neighborhood level predictors represented the actual experiences of both groups of women. A total of 1796 women met this criterion: 1580 whites and 216 blacks residing in 60 counties in the US. A flowchart describing the sample selection is available as a supplemental figure in supplementary materials available at http://dx.doi.org/10.1155/2013/490472. Counties were identified using the Federal Information Processing Standard (FIPS) code [42] to link county variables with cases' county of residence at diagnosis as recorded in the SEER dataset. 

### 2.2. Data Management: Sociodemographic Variables

Race was categorized as non-Hispanic white and non-Hispanic black. Marital status was categorized as married, widowed, divorced/separated, and never married. Employment status was categorized as women in the labor force and women not in the labor force. Education level was categorized as less than high school (<12 years), high school graduate (12 years), and at least some college (≥13 years). Age at diagnosis and income were analyzed as continuous variables; income was analyzed as inflation-adjusted annual household income (1990 standard).

*Clinical Variables*. Stage at presentation was categorized as in situ/localized, regional, and distant/unstaged. Surgical treatment was categorized as surgical treatment received, not received due to medical reasons, and not received due to nonmedical reasons. Radiation treatment was also categorized as received, not received due to medical reasons, and not received due to nonmedical reasons. Surgical or radiation treatment not received due to nonmedical reasons included those that were recommended but not performed, recommended and it was unknown if performed, or treatment information was unknown due to death certificate or autopsy diagnosis.

*Survival*. Survival time was calculated as the number of months between diagnosis and either date of death, date last known to be alive, or December 31, 2003. We created two censoring variables based on SEER cause of death variable: one that indicated if a person had died with breast cancer as the underlying cause, and the other that indicated if a person had died of any cause. Patients who died of other causes or were alive at the date of last followup were censored in the first censoring variable, and patients who were alive at the date of last followup were censored in the second censoring variable [13]. 

*Health Care Access Variables*.  Health care access was defined as the linear combination of measures of density of health care resources in the county [32]. We assessed specific variables related to health care resources at the county level and adjusted for differences in population size by dividing the counts per 10,000 people in each county. The derived variables were subject to principal component analysis (PCA) on count per 10,000 of number of hospitals, number of medical doctors, number of medical doctors with obstetrics and gynecology specialty, number of Osteopathic Doctors (DO), number of DOs with obstetrics and gynecology specialty, number of nurse practitioners, and number of mammography facilities. SAS Proc Factor was used to generate scores using an eigenequation [43] based on our input variables. The scores were categorized into tertiles:  poorest, middle, and highest. 

*Socioeconomic Status Variables*. County level SES was defined using the index of concentration in the extremes (ICE). This index was chosen over other approaches (such as the proportion of county residents below poverty level) because it allows for conceptualizing the concentration of affluence and disadvantage as falling along a single continuum. The index theoretically ranges from −1 (where all households are disadvantaged) through 0 (where there is equal proportion of affluent and disadvantaged households) to +1 (where all households are affluent) [44]. Two ICE indices were created: income based and education based. 

ICE-Income = ((# households with household income, $100,000+) − (# households with families below poverty level)/total number of households).

ICE-Education = ((% persons 25 years^+^ with 4^+^ yrs college) − (% persons 25 years^+^ with <9 yrs sch)/100).

For easy interpretation, these measures were also categorized into tertiles. To control for other county level variables that may be associated with income, we included county level proportion of blacks and percent non-English speaking [36]. 

*County Variables*. All county level variables were obtained from the Area Resource File for the year 2000 because it had the most complete year of data and straddled the years included in the SEER dataset. We compared scores for 1990 and 2000 and found them to be highly correlated (ICE income correlation coefficient = 0.90, *P* < 0.05 and ICE education correlation coefficient = 0.94,  *P* < 0.05). There were 3141 counties available for the county level analysis of health care access and SES. The first two components of the PCA had eigenvalues greater than 1, and the scree test also showed a clear break after the second component (figure not shown). Therefore, only the first two components were retained for further analysis. The two components together accounted for 50.5% of the total variance; components that had a factor loading value of greater than 0.4 were said to have loaded on a specific component. Based on this criterion, MDs per 10,000, MDs in Ob-Gyn per 10,000, nurse practitioners per 10,000, and DOs per 10,000 were loaded on the first component, hereafter named personnel. Number of hospitals per 10,000 and number of mammography facilities per 10,000 were loaded on the second component, hereafter named facilities. ICE-Income and ICE-Education scores were also calculated per county. 

## 3. Statistical Analysis

All statistical analyses were performed using SAS statistical software (SAS, Version 9.2). The analysis was weighted according to the US population size during the study period. Descriptive statistics were generated using chi-square statistics and *t*-tests. Multivariable proportional hazards regression was used to model the hazards of breast cancer and all-cause mortality in three separate models. The first model adjusted for demographic variables, the second model included clinical variables regarding stage of presentation and treatment, and the third model included county level variables. Robust sandwich estimates for the covariance matrix were used to account for the clustering of cases within counties, and the county FIPS code was specified as the clustering variable. 

## 4. Results

Table 1 provides information on the distribution of county variables relating to healthcare resources, socioeconomic status, and other controls across the US. 

Table 2 illustrates the distribution of demographic, clinical, and county level variables. On average, black patients were about 2 years younger compared with white patients, had a lower annual household income ($25,000 for blacks versus $44,700 for whites), were more likely to be single (19.1% of blacks versus 7.4% of whites) and divorced (19.7% of blacks versus 9.8% of whites), and were more likely to have less than a high school education (23.6% of blacks versus 9.5% of whites). The distribution of stage at diagnosis was similar between blacks and whites; about 53% of blacks were diagnosed at in situ/localized stage compared with 54.6% of white cases, and 14.2% of blacks were distant/unstaged compared with 13.3% of white cases. Black patients were also less likely to have received surgical (91% of blacks versus 95% of whites) and radiation treatment (31% of blacks versus 39% of whites) compared with white patients. 

At the county level, black patients were more likely than white patients to live in counties that had a higher proportion of households in poverty (36.9% of blacks versus 16.8% of whites), higher proportion of adults with less than 9 years of education (43.2% of blacks versus 26.9% of whites), and higher proportion of blacks (87.5% of blacks versus 67.5% of whites). Whites were more likely to reside in counties with the poorest healthcare facilities (38.1% of whites versus 32.7 of blacks) and a higher proportion of non-English speaking residents (77.6% of whites versus 59.7% of blacks). All of these differences were statistically significant at the  *P* < 0.05  level. 

In unadjusted analysis (Tables 3 and 4), black women had a 53% higher likelihood of dying of breast cancer (*P* = 0.008) and 32% higher likelihood of dying of any cause (*P* = 0.02) compared with white women. Having less than high school education increased the likelihood of breast cancer mortality by 68%, and all-cause mortality by 95%. Furthermore, being diagnosed at a distant stage and not receiving surgical or radiation treatment was also associated with significantly higher likelihood of death. 

Table 3 also presents the results of three sequential Cox Proportional Hazards multivariable models assessing the determinants of breast cancer survival. Model 1 assessed the effect of race on breast cancer mortality after adjusting for demographic variables. Race was no longer a statistically significant predictor of breast cancer death after adjusting for the individual variables (hazard ratio, 1.40; 95% CI, 0.99–1.97). Model 2 adjusted additionally for stage at presentation and treatment, and the effect of race remained nonsignificant. Model 3 presents the effect of county level variables adjusting for individual demographic and clinical variables on breast cancer mortality. Residing in counties with a higher proportion of households in poverty increased the likelihood of breast cancer deaths compared with counties with a higher proportion of affluent households. The hazard ratio of ICE-Income comparing the poorest versus highest group was 1.29 (95% CI, 0.82–2.05) and comparing the middle versus highest group was 1.49 (95% CI, 1.12–1.99). On the other hand, residing in counties with a higher proportion of residents with less than 9 years of education appeared to reduce the likelihood of breast cancer deaths. The hazard ratio of ICE-Education comparing the poorest versus highest group was 0.55 (95% CI, 0.31–0.98) and comparing the middle versus highest group was 0.65 (95% CI, 0.44–0.96). Furthermore, residing in a county with a higher proportion of black residents (≥6%) significantly increased the likelihood of breast cancer death (hazard ratio, 1.74; 95% CI, 1.21–2.48). Facilities and Personnel variables did not appear to have an independent significant effect on the likelihood of breast cancer death after adjusting for other variables in the model. 

Table 4 presents the results of the Cox Proportional Hazards models assessing the determinants of all-cause mortality. In contrast to the model predicting breast cancer survival (Table 3), race remained a statistically significant predictor of higher mortality among blacks compared to whites even after adjusting for individual demographic and clinical variables. In model 1 which adjusted for demographic variables, being black was associated with a 38% increase in the likelihood of death due to any cause compared with being white (95% CI, 1.08–1.76). In model 2, the hazard ratio associated with being black was 1.33 (95% CI, 1.04–1.70) after adjusting for demographic and clinical variables. After adjusting for county level variables in model 3, the effect of race was attenuated and became nonsignificant (hazard ratio, 1.27; 95% CI, 0.95–1.71). Personnel, Facilities, county proportion of blacks, and proportion of non-English speaking residents were not significantly associated with all-cause mortality. 

## 5. Discussion

In this study of black and white women with breast cancer, the effect of race on breast cancer mortality became nonsignificant after adjusting for individual demographic variables. For all-cause mortality, race was a significant predictor even after adjusting for demographic and clinical variables such as stage of presentation and treatment. 

We observed sustained differences in the receipt of surgical and radiation treatment between black and white patients in this study. This is particularly troubling given that these treatment variables remained the most significant predictors of breast cancer mortality and all-cause mortality even after adjusting for individual and neighborhood level variables. It has been suggested that the disparity in treatment receipt among black women may be due to cultural factors which make them more likely to refuse treatment. However, in this study we were able to distinguish between patients who did not receive treatment due to medical reasons or nonmedical reasons. Among women who did not receive treatment due to medical reasons (implying that a medical personnel made the decision not to treat), the proportion of black women was still much higher. More research is urgently needed in this area to better understand the factors that determine who gets treatment.

Upon adjusting for county level healthcare access variables, race was no longer significantly associated with all-cause mortality. Facilities and personnel variables did not appear to have a significant independent effect on the likelihood of breast cancer or all-cause mortality after adjusting for other variables in the model. Furthermore, residing in counties with a higher proportion of households in poverty increased the likelihood of breast cancer mortality and all-cause mortality compared with counties with a higher proportion of affluent households. 

Our findings suggest that neighborhood poverty and lack of healthcare resources to care might explain part of the black-white disparity in breast cancer survival especially if examined from both individual and neighborhood levels. Many studies have sought to explain the causes of racial disparities in breast cancer survival [10–13, 17, 23, 45–54], with varying results. However, none of these studies assessed the role of neighborhood healthcare resources in breast cancer or all-cause survival although other studies have been published about the impact of neighborhood healthcare resources on stage at diagnosis [12, 33, 55]. Our study suggests that stage at presentation is an important predictor of breast cancer survival; late stage at diagnosis was associated with a fourfold increase in the hazard of breast cancer mortality after adjusting for other variables. Studies that assessed the role of county level healthcare access on late stage diagnosis of cancer found that women residing in counties with fewer physicians [33] and poor access to mammography facilities [32] were more likely to have late stage cancer diagnosis. Other studies have suggested that important predictors of mammography use are having a primary care physician, travel times, and public transportation hassles [55–64]. These are all factors which may be compounded if there are inadequate healthcare facilities and personnel within a county.

We anticipated that these county characteristics, number of physicians, and mammography facilities would also be associated with breast cancer survival through the availability of early diagnosis and adequate treatment. However, our measures of healthcare access did not independently predict breast cancer survival, even though the initial racial disparity in survival disappeared. This finding is consistent with a recent publication which did not find an association between the availability of medical resources and breast cancer mortality at the county level [65]. Our finding may be due to several factors. First is the geographic level at which the neighborhood attributes are being measured. It is likely that some heterogeneity in exposure (i.e., county SES and healthcare access) is lost by aggregating neighborhood measures to the county level as opposed to the zip code or census tract level. However, due to patient privacy concerns, the SEER dataset does not routinely disclose patient geographic location at levels smaller than the county. Second, this study accounted for the availability of healthcare resources but not accessibility. However, the concept of healthcare access is very complex and multidimensional, incorporating aspects of availability such as the presence of medical facilities and personnel as well as aspects of accessibility such as distance, affordability, and cultural barriers. This study serves as a first step in understanding the role of one aspect of healthcare access on breast cancer survival, and future studies may build on this research to further improve understanding of other aspects. 

Third, other neighborhood level factors such as racial or economic segregation may also be important in understanding racial differences in breast cancer survival and need to be examined. For instance, we found that women residing in counties with a higher proportion of blacks had significantly higher hazards of breast cancer mortality. This is especially relevant to this study of racial disparities because studies have shown that due to lower income levels on average, blacks are more likely to reside in poor counties [26, 32]. However, due to established social and family networks, even blacks that belong to higher SES groups are more likely to continue to reside in these poor counties. This has implications for the understanding of the impact of socioeconomic status and breast cancer survival among blacks, because black women earning higher incomes may not benefit as much from their socioeconomic status as white women earning similar wages but residing in high SES counties. 

We observed a significant increase hazard of breast cancer and all-cause mortality due to neighborhood income. Women residing in counties with a higher proportion of low income residents compared with high income residents had higher hazards. This is consistent with other studies using other definitions of county SES [20, 23, 66, 67]. However, we also observed significant reduction in the hazard of breast cancer and all-cause mortality for counties with a higher proportion of less educated residents. This observation was unexpected especially since higher individual education level was found to be protective in this study as well as in others [24, 37, 66, 68]. One explanation for this finding may be the small sample size in counties with a higher proportion of less educated residents. Another potential explanation is the high proportion of immigrants who may be less educated but more likely to have close-knit social networks that has been found to be protective against adverse health outcomes [69]. 

The major strength of this study was the availability of individual SES information for cancer patients. This allowed for better control of potential confounding of the association between neighborhood characteristics and survival by individual SES. Another strength is the development of a specific measure of neighborhood healthcare access using the availability of healthcare resources including mammography facilities to better capture factors that may affect early detection which is a major determinant of survival. 

There are several limitations of our study. First, there is a possibility of exposure misclassification bias because measures of individual SES (household income, education, and employment) were not obtained at the time of diagnosis with cancer. These measures were obtained through surveys which may have been administered before or after cancer diagnosis. However, our analysis was restricted to breast cancer cases ages 40 and older, reducing the likelihood of dramatic changes in SES through the study period. Secondly, our measure of healthcare access at the county level is an indicator of availability, not necessarily accessibility. Several other factors may determine if a person actually benefits from living in a county with good healthcare facilities such as language or cultural barriers, mistrust of the medical system, and lack of health insurance. Thirdly, for analytical reasons, the study sample was restricted to women residing in counties that had at least one black and one white breast cancer patients. This implies that the population may be more urban compared with the rest of the US. However, the results of this analysis may still be applicable to semi-urban or rural areas where the impact of county SES and healthcare access on breast cancer survival may be even more pronounced. 

We performed a sensitivity analysis using only white women (*n* = 1580) and found similar results; the neighborhood variables did not significantly predict breast cancer survival among white women. However they did attenuate the effect of stage and treatment on survival. This supports our earlier conclusion that the county level variables may influence breast cancer survival (for both white and black women) through the availability of screening and timely treatment. The differential impact of these county variables which may contribute to the racial disparities in breast cancer survival between black and white women (effect measure modification) was assessed through interaction terms in the final model; however there were no significant interactions possibly due to the low sample size of black women within each group. 

Finally, we performed a post hoc power analysis to assess the statistical power of the study to detect a statistically significant difference between breast cancer survival in black and white women. Given the sample size of 216 black and 1580 white patients, type 1 error rate of 0.05, and a followup of 30 years, the study had a power level of 85%. This implies that if a difference in races existed, we had an 85% chance of detecting it. 

In summary, our study adds an important component to the existing evidence on survival disparities by conceptualizing healthcare access at the county level as a potential determinant of health outcomes and as a potential modifier of the association between race and mortality. Further studies may focus on defining healthcare access at smaller geographic levels, for example, zip code or census tracts which may be more homogenous in the distribution of healthcare facilities and personnel. Furthermore, while our study focused on the quantity of healthcare facilities and personnel, other studies may attempt to include a measure of healthcare quality as another measure of healthcare access and a potential determinant of survival.

## Supplementary Material

Supplemental figure narrative: The analysis was based on breast cancer survival among non-Hispanic black and white women ages 40 and older from the SEER-NLMS dataset that had a valid match with the National Death Index for vital status. We further required that women reside in a county that included at least 1 black and 1 white breast cancer case to facilitate valid inferences about the impact of county level variables on survival by race. A total of 1580 women fulfilled all our study criteria; 216 black and 1580 white women.

## Figures and Tables

**Table 1:** Descriptive statistics of healthcare access and socio-economic characteristics of 3141 US counties in 2000-2001, Area Resource File 2009-2010.

Variable	All US counties (*n* = 3141)				Analysis counties (*n* = 60)			
	Mean	Std Dev	Min	Max	Mean	Std Dev	Min	Max
County level health care access								

^ a^Personnel	−2.058*E* − 16	1.000	−1.519	13.777	0.814	0.866	−0.819	3.054
^ a^Facilities	−1.968*E* − 16	1.000	−2.565	10.801	−0.482	0.316	−1.014	0.605

County level income and education								

^ b^Index of concentration at the extremes (Income)	−0.009	0.076	−0.429	0.358	0.067	0.098	−0.168	0.359
^ b^Index of concentration at the extremes (Education)	0.074	0.114	−0.394	0.619	0.160	0.122	−0.137	0.390

County level controls								

% Non-English speaking	1.631	2.610	0	28.715	4.970	4.405	0	15.889
% Black	8.763	14.512	0	86.500	16.06	15.49	0.300	67.300

^a^Personnel and facilities, two measures of health care access were defined using principal components analysis on the count per 10,000 population of county level variables. Facilities: hospitals, mammography facilities; Personnel: MDs, Dos, and nurse practitioners.

^b^Measures of SES at the county level are the ICE-Income and ICE-Education variables, defined as

ICE-Education = (% 25^+^ years with college degree − % 25^+^ years with <9 yrs education).

ICE-Income = (% with HH income > $100,000 − % HH in poverty).

**Table 2:** Distribution of individual and county-level characteristics of breast cancer cases by race.

Characteristic	Black (*N* = 216) %	White (*N* = 1580) %	*P* value
Age at diagnosis mean (SD)	61.14 (14.34)	63.51 (13.49)	0.01
Income/$1000 mean (SD)	25.03 (24.38)	44.72 (40.40)	<0.001
Marital Status			
Single	19.11	7.36	
Married	28.60	53.55	
Widowed	30.19	28.04	<0.001
Divorced/separated	19.72	9.84	
Missing	2.39	1.21	
Employment			
In labor force	49.91	47.66	
Not in labor force	46.10	48.18	0.79
Missing	3.99	4.17	
Education			
<High school	23.57	9.51	
High school graduate	29.99	38.76	<0.001
College	46.44	51.73	
Rural/urban			
Rural	6.22	9.15	0.12
Urban	93.78	90.85	
Stage of diagnosis			
In situ/localized	53.01	54.62	0.96
Regional	27.69	27.11	
Distant/unstaged	14.16	13.25	
Missing	5.14	5.02	
Surgical treatment			
Received	91.23	95.07	
None, medical reasons	4.30	2.06	0.03
None-non medical reasons	4.47	2.87	
Radiation treatment			
Received	31.17	38.61	
None, medical reasons	62.42	55.68	0.07
None-nonmedical reasons	6.41	5.70	
Income disparity			
Poorest	36.94	16.77	
Middle	30.79	34.87	<0.001
Highest	32.28	48.36	
Education disparity			
Poorest	43.20	26.90	
Middle	24.69	31.43	<0.001
Highest	32.11	41.67	
Facilities			
Poorest	32.69	38.10	
Middle	50.57	38.46	0.0009
Highest	16.74	23.44	
Personnel			
Poorest	13.09	12.10	
Middle	45.67	44.28	0.76
Highest	41.24	43.62	
Percent non-English speaking			
<3%	40.32	22.42	<0.001
≥3%	59.68	77.58	
Percent black			
<6%	12.48	32.49	<0.001
≥6%	87.52	67.51	

^a^Personnel and facilities, two measures of health care access were defined using principal components analysis on the count per 10,000 population of county level variables and then categorized into tertiles. Facilities: hospitals, mammography facilities; Personnel: MDs, Dos, and nurse practitioners.

^b^Measures of SES at the county level are the ICE-Income and ICE-Education variables which were calculated and categorized into tertiles, defined as:

ICE-Education = (% 25^+^ years with college degree − % 25^+^ years with <9 yrs education).

ICE-Income = (% with HH income >$100,000 − % HH in poverty).

**Table 3:** Cox Proportional Hazard analysis of breast cancer mortality, SEER-NLMS, 1973–2003.

Characteristics	Hazard ratio (95% CI) of breast cancer mortality			
	Unadjusted	Model 1 Demographics^a^	Model 2 +Clinical^b^	Model 3 +County^c^
Race				
Black	1.53 (1.11–2.11)**	1.40 (0.99–1.97)	1.40 (0.99–1.98)	1.32 (0.73–2.41)
White (ref.)	1.00	1.00	1.00	1.00
Age	1.02 (1.01-1.02)**	1.01 (1.00–1.02)	1.01 (0.99–1.02)	1.01 (0.99–1.02)
Income/$1000	0.99 (0.99-1.00)*	0.99 (0.99-1.00)	0.99 (0.99–1.00)	0.99 (0.99–1.00)
Employed				
Not in labor force	1.09 (0.87–1.38)	1.02 (0.78–1.33)	1.33 (1.01–1.74)*	1.38 (0.89–2.14)
In labor force (ref.)	1.00	1.00	1.00	1.00
Marital status				
Single	1.35 (0.90–2.03)	1.10 (0.72–1.70)	1.21 (0.78–1.87)	1.16 (0.67–2.02)
Divorced/separated	1.57 (1.12–2.19)	1.43 (1.01–2.03)*	1.57 (1.09–2.25)*	1.64 (1.09–2.45)*
Widowed	1.05 (0.79–1.38)	0.95 (0.71–1.28)	1.09 (0.80–1.48)	1.09 (0.72–1.66)
Married (ref.)	1.00	1.00	1.00	1.00
Education				
<High school	1.68 (1.19–2.36)**	1.44 (1.00–2.05)*	1.36 (0.95–1.94)	1.35 (0.99–1.85)
High school grad	1.43 (1.12–1.83)**	1.38 (1.08–1.78)*	1.42 (1.09–1.83)**	1.55 (1.22–1.96)**
College (ref.)	1.00	1.00	1.00	1.00
Stage of presentation				
Regional	4.48 (3.23–6.22)**		3.27 (2.44–4.39)***	3.38 (2.48–4.60)***
Distant/unstaged	9.59 (6.88–13.39)**		4.18 (3.03–5.77)***	5.78 (3.06–10.93)***
In situ/local (ref.)	1.00		1.00	1.00
Surgical treatment				
Nonemedical reasons	16.66 (11.28–24.59)**		8.55 (5.54–13.21)***	8.15 (3.56–18.68)***
None-non medical reasons	5.07 (3.36–7.60)**		3.72 (2.39–5.84)***	3.23 (1.56–6.68)**
Received (ref.)	1.00		1.00	1.00
Radiation treatment				
None-autopsy diagnosis	1.05 (0.82–1.35)		0.87 (0.67–1.41)	0.85 (0.62–1.16)
None-refused/unknown	2.57 (1.68–3.91)**		2.10 (1.35–3.28)**	1.91 (0.95–3.86)
Received (ref.)	1.00		1.00	1.00
Rural/urban				
Urban	1.12 (0.74–1.69)			1.40 (0.84–2.35)*
Rural (ref.)	1.00			1.00
^ d^Income disparity				
Poorest	1.24 (0.96–1.61)			1.29 (0.82–2.05)
Middle	1.17 (0.86–1.59)			1.49 (1.12–1.99)**
Highest (ref.)	1.00			1.00
^ d^Education disparity				
Poorest	1.03 (0.74–1.43)			0.55 (0.31–0.98)*
Middle	1.03 (0.77–1.39)			0.65 (0.44–0.96)*
Highest (ref.)	1.00			1.00
^ e^Facilities				
Poorest	0.76 (0.59–0.97)			1.01 (0.61–1.68)
Middle	1.00 (0.77–1.31)			1.15 (0.66–1.99)
Highest (ref.)	1.00			1.00
^ e^Personnel				
Poorest	1.12 (0.78–1.61)			0.78 (0.40–1.51)
Middle	0.96 (0.72–1.27)			0.78 (0.49–1.24)
Highest (ref.)	1.00			1.00
Proportion non-English speaking				
≥3%	0.75 (0.59–0.95)*			0.91 (0.56–1.48)
<3% (ref.)	1.00			1.00
Proportion black				
≥6%	1.29 (0.98–1.68)			1.74 (1.21–2.48)**
<6% (ref.)	1.00			1.00

*P < 0.05, **P < 0.01, ***P < 0.001; CI: confidence interval; ref: reference group.

^a^Model adjusting for individual demographic variables only.

^b^Model adjusting for clinical variables such as stage at presentation and treatment in addition to demographic variables.

^c^Model adjusting for county level variables including healthcare access and SES in addition to individual demographics and clinical variables.

^d^Measures of SES at the county level are the ICE-Income and ICE-Education variables which were calculated and categorized into tertiles, defined as

ICE-Education = (% 25^+^ years with college degree − %25^+^ years with <9 yrs education).

ICE-Income = (% with HH income > $100,000 − % HH in poverty).

^e^Personnel and facilities, two measures of health care access were defined using principal components analysis on the count per 10,000 population of county level variables and then categorized into tertiles. Facilities: hospitals, mammography facilities; Personnel: MDs, Dos, and nurse practitioners.

**Table 4:** Cox Proportional Hazard analysis of all-cause mortality, SEER-NLMS, 1973–2003.

Characteristics	Hazard ratio (95% CI) of all-cause mortality			
	Unadjusted	Model 1 Demographics^a^	Model 2 +Clinical^b^	Model 3 +County^c^
Race				
Black	1.32 (1.05–1.66)*	1.38 (1.08–1.76)**	1.33 (1.04–1.70)*	1.27 (0.95–1.71)
White (ref.)	1.00	1.00	1.00	1.00
Age	1.06 (1.05-1.06)***	1.05 (1.04–1.06)***	1.05 (1.04-1.05)***	1.05 (1.03–1.06)***
Income/$1000	0.99 (0.98-0.99)***	0.99 (0.99-1.00)	0.99 (0.99-1.00)	0.99 (0.99-1.00)
Employed				
Not in labor force	1.60 (1.36–1.88)***	1.0 (0.83–1.21)	1.18 (0.98–1.43)	1.18 (0.92–1.51)
In labor force (ref.)	1.00	1.00	1.00	1.00
Marital status				
Single	1.48 (1.11–1.97)***	1.32 (0.97–1.78)	1.44 (1.06–1.95)*	1.40 (0.95–2.06)
Divorced/separated	1.39 (1.08–1.79)***	1.39 (1.07–1.81)*	1.58 (1.21–2.07)**	1.59 (1.19–2.14)**
Widowed	1.70 (1.43–2.02)**	1.16 (0.96–1.39)	1.29 (1.07–1.57)**	1.36 (1.01–1.83)*
Married (ref.)	1.00	1.00	1.00	1.00
Education				
<High school	1.95 (1.56–2.42)**	1.49 (1.19–1.87)**	1.44 (1.14–1.79)**	1.41 (1.05–1.89)*
High school grad	1.32 (1.12–1.56)***	1.23 (1.04–1.46)*	1.28 (1.08–1.52)**	1.34 (1.10–1.64)**
College (ref.)	1.00	1.00	1.00	1.00
Stage of presentation				
Regional	1.72 (1.43–2.07)***		1.68 (1.40–2.02)***	1.69 (1.41–2.04)***
Distant/unstaged	3.29 (2.71–4.02)***		1.87 (1.51–2.30)***	2.16 (1.55–3.02)***
In situ/local (ref.)	1.00		1.00	1.00
Surgical treatment				
None-medical reasons	11.08 (7.98–15.38)***		7.35 (5.14–10.52)***	6.79 (3.79–12.15)***
None-non medical reasons	3.61 (2.65–4.91)***		2.96 (2.12–4.13)***	2.81 (1.64–4.80)***
Received (ref.)	1.00		1.00	1.00
Radiation treatment				
None-medical reasons	1.25 (1.05–1.49)***		0.96 (0.79–1.15)	0.95 (0.77–1.18)
None-non medical reasons	2.46 (1.80–3.35)***		2.23 (1.61–3.09)**	2.10 (1.46–3.03)***
Received (ref.)			1.00	1.00
Rural/Urban				
Urban	1.29 (0.96–1.74)			1.28 (0.87–1.88)
Rural (ref.)	1.00			1.00
^ d^Income disparity				
Poorest	1.27 (1.05–1.53)**			1.37 (1.03–1.82)*
Middle	1.12 (0.88–1.41)			1.27 (1.03–1.58)*
Highest (ref.)	1.00			1.00
^ d^Education Disparity				
Poorest	1.06 (0.82–1.39)			0.61 (0.47–0.81)**
Middle	1.10 (0.87–1.39)			0.75 (0.60–0.93)**
Highest (ref.)	1.00			1.00
^ e^Facilities				
Poorest	0.82 (0.69–0.98)**			0.98 (0.68–1.41)
Middle	1.12 (0.90–1.40)			1.13 (0.79–1.64)
Highest (ref.)	1.00			1.00
^ e^Personnel				
Poorest	1.09 (0.81–1.47)			0.93 (0.64–1.36)
Middle	1.09 (0.87–1.36)			0.98 (0.79–1.21)
Highest (ref.)	1.00			1.00
Proportion non-English speaking				
>3%	0.76 (0.63–0.93)**			0.87 (0.63–1.19)
<3% (ref.)	1.00			1.00
Proportion black				
>6%	1.01 (0.82–1.25)			1.19 (0.97–1.47)
<6% (ref.)	1.00			1.00

*P < 0.05, **P < 0.01, ***P < 0.001; CI: confidence interval; ref: reference group

^a^Model adjusting for individual demographic variables only.

^b^Model adjusting for clinical variables such as stage at presentation and treatment in addition to demographic variables.

^c^Model adjusting for county level variables including healthcare access and SES in addition to individual demographics and clinical variables.

^d^Measures of SES at the county level are the ICE-Income and ICE-Education variables which were calculated and categorized into tertiles, defined as

ICE-Education = (% 25^+^ years with college degree − % 25^+^ years with <9 yrs education).

ICE-Income = (% with HH income > $100,000 − % HH in poverty).

^e^Personnel and Facilities, two measures of health care access were defined using principal components analysis on the count per 10,000 population of county level variables and then categorized into tertiles. Facilities: hospitals, mammography facilities; Personnel: MDs, Dos, and nurse practitioners.

## References

[1] Martin JA, Hamilton BE, Sutton PD, Ventura SJ, Menacker F, Kirmeyer S (2006). Births: final data for 2004. *National Vital Statistics Reports*.

[2] Smigal C, Jemal A, Ward E (2006). Trends in breast cancer by race and ethnicity: update 2006. *CA: A Cancer Journal for Clinicians*.

[3] Glass AG, Lacey JV, Carreon JD, Hoover RN (2007). Breast cancer incidence, 1980–2006: combined roles of menopausal hormone therapy, screening mammography, and estrogen receptor status. *Journal of the National Cancer Institute*.

[4] Krieger N, Chen JT, Waterman PD (2010). Decline in US breast cancer rates after the women’s health initiative: socioeconomic and racial/ethnic differentials. *American Journal of Public Health*.

[5] Jemal A, Ward E, Thun MJ (2007). Recent trends in breast cancer incidence rates by age and tumor characteristics among U.S. women. *Breast Cancer Research*.

[6] Hausauer AK, Keegan TH, Chang ET, Clarke CA (2007). Recent breast cancer trends among Asian/Pacific Islander, Hispanic, and African-American women in the US: changes by tumor subtype. *Breast Cancer Research*.

[7] Li CI, Daling JR (2007). Changes in breast cancer incidence rates in the United States by histologic subtype and race/ethnicity, 1995 to 2004. *Cancer Epidemiology Biomarkers and Prevention*.

[8] Howlader N, Noone AM, Krapcho M, Neyman N, Aminou R, Altekruse SF (2012). *SEER Cancer Statistics Review, 1975–2009 (Vintage 2009 Populations)*.

[9] American Cancer Society (2012). *Cancer Facts & Figures*.

[10] Newman LA (2005). Breast cancer in African-American women. *Oncologist*.

[11] Balasubramanian BA, Demissie K, Crabtree BF, Strickland PAO, Kohler B, Rhoads GG (2010). Racial differences in adjuvant systemic therapy for early breast cancer among medicaid beneficiaries. *Breast Journal*.

[12] Celaya MO, Berke EM, Onega TL (2010). Breast cancer stage at diagnosis and geographic access to mammography screening (New Hampshire, 1998–2004). *Rural and Remote Health*.

[13] Du XL, Lin CC, Johnson NJ, Altekruse S (2011). Effects of individual-level socioeconomic factors on racial disparities in cancer treatment and survival: findings from the national longitudinal mortality study, 1979–2003. *Cancer*.

[14] Freedman RA, Virgo KS, He Y (2011). The association of race/ethnicity, insurance status, and socioeconomic factors with breast cancer care. *Cancer*.

[15] Deshpande AD, Jeffe DB, Gnerlich J, Iqbal AZ, Thummalakunta A, Margenthaler JA (2009). Racial disparities in breast cancer survival: an analysis by age and stage. *Journal of Surgical Research*.

[16] Booth CM, Li G, Zhang-Salomons J, Mackillop WJ (2010). The impact of socioeconomic status on stage of cancer at diagnosis and survival: a population-based study in Ontario, Canada. *Cancer*.

[17] Du XL, Fang S, Meyer TE (2008). Impact of treatment and socioeconomic status on racial disparities in survival among older women with breast cancer. *American Journal of Clinical Oncology*.

[18] Harper S, Lynch J, Meersman SC, Breen N, Davis WW, Reichman MC (2009). Trends in area-socioeconomic and race-ethnic disparities in breast cancer incidence, stage at diagnosis, screening, mortality, and survival among women ages 50 years and over (1987–2005). *Cancer Epidemiology Biomarkers and Prevention*.

[19] Komenaka IK, Martinez ME, Pennington RE, Hsu CH, Clare SE, Thompson PA (2010). Race and ethnicity and breast cancer outcomes in an underinsured population. *Journal of the National Cancer Institute*.

[20] Niu X, Pawlish KS, Roche LM (2010). Cancer survival disparities by race/ethnicity and socioeconomic status in New Jersey. *Journal of Health Care for the Poor and Underserved*.

[21] Martin DN, Boersma BJ, Yi M (2009). Differences in the tumor microenvironment between African-American and European-American breast cancer patients. *PLoS ONE*.

[22] Braun KL, Fong M, Gotay C, Pagano IS, Chong C (2005). Ethnicity and breast cancer in Hawaii: increased survival but continued disparity. *Ethnicity and Disease*.

[23] Schootman M, Jeffe DB, Gillanders WE, Aft R (2009). Racial disparities in the development of breast cancer metastases among older women. *Cancer*.

[24] Byers TE, Wolf HJ, Bauer KR (2008). The impact of socioeconomic status on survival after cancer in the United States: findings from the national program of cancer registries patterns of care study. *Cancer*.

[25] Albain KS, Unger JM, Crowley JJ, Coltman CA, Hershman DL (2009). Racial disparities in cancer survival among randomized clinical trials patients of the southwest oncology group. *Journal of the National Cancer Institute*.

[26] Warner ET, Gomez SL (2010). Impact of neighborhood racial composition and metropolitan residential segregation on disparities in breast cancer stage at diagnosis and survival between black and white women in California. *Journal of Community Health*.

[27] Newman LA, Griffith KA, Jatoi I, Simon MS, Crowe JP, Colditz GA (2006). Meta-analysis of survival in African American and white American patients with breast cancer: ethnicity compared with socioeconomic status. *Journal of Clinical Oncology*.

[28] Taioli E, Attong-Rogers A, Layne P, Roach V, Ragin C (2010). Breast cancer survival in women of African descent living in the US and in the Caribbean: effect of place of birth. *Breast Cancer Research and Treatment*.

[29] Chang SW, Kerlikowske K, Napoles-Springer A, Posner SF, Sickles EA, Perez-Stable EJ (1996). Racial differences in timeliness of follow-up after abnormal screening mammography. *Cancer*.

[30] Elmore JG, Nakano CY, Linden HM, Reisch LM, Ayanian JZ, Larson EB (2005). Racial inequities in the timing of breast cancer detection, diagnosis, and initiation of treatment. *Medical Care*.

[31] Jones BA, Dailey A, Calvocoressi L (2005). Inadequate follow-up of abnormal screening mammograms: findings from the race differences in screening mammography process study (United States). *Cancer Causes and Control*.

[32] Dai D (2010). Black residential segregation, disparities in spatial access to health care facilities, and late-stage breast cancer diagnosis in metropolitan Detroit. *Health and Place*.

[33] Coughlin SS, Richardson LC, Orelien J, Thompson T, Richards TB, Sabatino SA (2009). Contextual analysis of breast cancer stage at diagnosis among women in the United States, 2004. *The Open Health Services and Policy Journal*.

[34] Coughlin SS, Leadbetter S, Richards T, Sabatino SA (2008). Contextual analysis of breast and cervical cancer screening and factors associated with health care access among United States women, 2002. *Social Science and Medicine*.

[35] Ricciardi R, Roberts PL, Read TE, Baxter NN, Marcello PW, Schoetz DJ (2011). Presence of specialty surgeons reduces the likelihood of colostomy after proctectomy for rectal cancer. *Diseases of the Colon and Rectum*.

[36] Benjamins MR, Kirby JB, Huie SAB (2004). County characteristics and racial and ethnic disparities in the use of preventive services. *Preventive Medicine*.

[37] Clegg LX, Reichman ME, Miller BA (2009). Impact of socioeconomic status on cancer incidence and stage at diagnosis: selected findings from the surveillance, epidemiology, and end results: national longitudinal mortality study. *Cancer Causes and Control*.

[38] National Cancer Institute National Longitudinal Mortality Survey (NLMS) and Linked SEER-NLMS Databases. http://surveillance.cancer.gov/disparities/nlms/.

[39] National Cancer Institute Surveillance Epidemiology and End Results. http://seer.cancer.gov/index.html.

[40] US Department of Health and Human Services Health Resources and Services Administration Area Resource File. http://arf.hrsa.gov/index.htm.

[41] Food and Drug Administration Mammography Quality Standards Act and Program. http://www.fda.gov/Radiation-EmittingProducts/MammographyQualityStandardsActandProgram/default.htm.

[42] U.S. Department of Commerce National Bureau of Standards Counties and Equivalent Entities of the United States, Its Possessions, and Associated Areas. http://www.itl.nist.gov/fipspubs/fip6-4.htm.

[43] Vyas S, Kumaranayake L (2006). Constructing socio-economic status indices: how to use principal components analysis. *Health Policy and Planning*.

[44] Carpiano RM, Lloyd JEV, Hertzman C (2009). Concentrated affluence, concentrated disadvantage, and children’s readiness for school: a population-based, multi-level investigation. *Social Science and Medicine*.

[45] Braithwaite D, Tammemagi CM, Moore DH (2009). Hypertension is an independent predictor of survival disparity between African-American and white breast cancer patients. *International Journal of Cancer*.

[46] Campbell RT, Li X, Dolecek TA, Barrett RE, Weaver KE, Warnecke RB (2009). Economic, racial and ethnic disparities in breast cancer in the US: towards a more comprehensive model. *Health and Place*.

[47] Menashe I, Anderson WF, Jatoi I, Rosenberg PS (2009). Underlying causes of the black-white racial disparity in breast cancer mortality: a population-based analysis. *Journal of the National Cancer Institute*.

[48] Haas JS, Earle CC, Orav JE (2008). Racial segregation and disparities in breast cancer care and mortality. *Cancer*.

[49] Kim SH, Ferrante J, Won BR, Hameed M (2008). Barriers to adequate follow-up during adjuvant therapy may be important factors in the worse outcome for Black women after breast cancer treatment. *World Journal of Surgical Oncology*.

[50] Demicheli R, Retsky MW, Hrushesky WJM, Baum M, Gukas ID, Jatoi I (2007). Racial disparities in breast cancer outcome: insights into host-tumor interactions. *Cancer*.

[51] Griggs JJ, Sorbero MES, Stark AT, Heininger SE, Dick AW (2003). Racial disparity in the dose and dose intensity of breast cancer adjuvant chemotherapy. *Breast Cancer Research and Treatment*.

[52] Gregorio DI, Walsh SJ, Tate JP (1999). Diminished socioeconomic and racial disparity in the detection of early-stage breast cancer, Connecticut, 1986–1995. *Ethnicity and Disease*.

[53] Schootman M, Jeffe DB, Lian M, Gillanders WE, Aft R (2009). The role of poverty rate and racial distribution in the geographic clustering of breast cancer survival among older women: a geographic and multilevel analysis. *American Journal of Epidemiology*.

[54] Bao Y, Fox SA, Escarce JJ (2007). Socioeconomic and racial/ethnic differences in the discussion of cancer screening: “between-” versus “within-” physician differences. *Health Services Research*.

[55] Tarlov E, Zenk SN, Campbell RT, Warnecke RB, Block R (2009). Characteristics of mammography facility locations and stage of breast cancer at diagnosis in Chicago. *Journal of Urban Health*.

[56] Gerend MA, Pai M (2008). Social determinants of black-white disparities in breast cancer mortality: a review. *Cancer Epidemiology Biomarkers and Prevention*.

[57] Adams SA, Smith ER, Hardin J, Prabhu-Das I, Fulton J, Hebert JR (2009). Racial differences in follow-up of abnormal mammography findings among economically disadvantaged women. *Cancer*.

[58] Jackson MC, Davis WW, Waldron W, McNeel TS, Pfeiffer R, Breen N (2009). Impact of geography on mammography use in California. *Cancer Causes & Control*.

[59] Dailey AB, Kasl SV, Holford TR, Calvocoressi L, Jones BA (2007). Neighborhood-level socioeconomic predictors of nonadherence to mammography screening guidelines. *Cancer Epidemiology Biomarkers and Prevention*.

[60] Makuc DM, Breen N, Meissner HI, Vernon SW, Cohen A (2007). Financial barriers to mammography: who pays out-of-pocket?. *Journal of Women’s Health*.

[61] Rosenberg L, Wise LA, Palmer JR, Horton NJ, Adams-Campbell LL (2005). A multilevel study of socioeconomic predictors of regular mammography use among African-American women. *Cancer Epidemiology Biomarkers and Prevention*.

[62] Kothari AR, Birch S (2004). Individual and regional determinants of mammography uptake. *Canadian Journal of Public Health*.

[63] O’Malley MS, Earp JAL, Hawley ST, Schell MJ, Mathews HF, Mitchell J (2001). The association of race/ethnicity, socioeconomic status, and physician recommendation for mammography: who gets the message about breast cancer screening?. *American Journal of Public Health*.

[64] Hyndman JCG, Holman CDJ (2000). Differential effects on socioeconomic groups of modelling the location of mammography screening clinics using geographic information systems. *Australian and New Zealand Journal of Public Health*.

[65] Pisu M, Wang D, Martin MY, Baltrus P, Levine RS (2010). Presence of medical schools may contribute to reducing breast cancer mortality and disparities. *Journal of Health Care for the Poor and Underserved*.

[66] Sprague BL, Trentham-Dietz A, Gangnon RE, Ramchandani R, Hampton JM, Robert SA (2011). Socioeconomic status and survival after an invasive breast cancer diagnosis. *Cancer*.

[67] Yu XQ (2009). Socioeconomic disparities in breast cancer survival: relation to stage at diagnosis, treatment and race. *BMC Cancer*.

[68] Hussain SK, Altieri A, Sundquist J, Hemminki K (2008). Influence of education level on breast cancer risk and survival in Sweden between 1990 and 2004. *International Journal of Cancer*.

[69] Keegan THM, Quach T, Shema S, Glaser SL, Gomez SL (2010). The influence of nativity and neighborhoods on breast cancer stage at diagnosis and survival among California Hispanic women. *BMC Cancer*.

