# Supplementary material for: Individual and Neighborhood Socioeconomic Status and Healthcare Resources in Relation to Black-White Breast Cancer Survival Disparities
Source: J Cancer Epidemiol. 2013 Feb 20;2013:490472. doi: 10.1155/2013/490472 (PMC3590635; doi:10.1155/2013/490472)
Supplement: Supplementary file 1 — Supplemental figure narrative: The analysis was based on breast cancer survival among non-Hispanic black and white women ages 40 and older from the SEER-NLMS dataset that had a valid match with the National Death Index for vital status. We further required that women reside in a county that included at least 1 black and 1 white breast cancer case to facilitate valid inferences about the impact of county level variables on survival by race. A total of 1580 women fulfilled all our study criteria; 216 black and 1580 white women. [file 490472.f1.docx]

Reside in county with at least 1 black, 1 white case (N=1796)

First Primary Breast Cancer (N=3511)

Cases =>40 years (N=4117)

Cases <40 years (N=268)

Black and White cases (N=4385)

Other races (N=381)

Hispanic cases (N=1188)

Non-Hispanic cases (N=4,776)

Breast Cancer cases (N=5,964)

Non Breast Cancer cases (N=12,318)

Females only (N=18,281)

Males (N=17,113)

Valid Records for National Death Index Match (N=35,394)

216 Black cases

1580 White cases

Supplemental Figure 1: Flowchart of final sample selection

SEER-NLMS Linked Dataset (N=35,839)

Invalid Records (N=445)
